# Sex differences in ventricular-vascular interactions associated with aerobic capacity

**DOI:** 10.1186/s44156-024-00066-9

**Published:** 2025-01-20

**Authors:** Barbara N. Morrison, Peter M. Mittermaier, Garth R. Lester, Michael E. Bodner, Anita T. Cote

**Affiliations:** 1 School of Human Kinetics, Trinity Western University, CANIL Building, Rm. 115 22500 University Drive, Langley, BC V2Y 1Y1 Canada; 2 Faculty of Natural & Applied Sciences, Trinity Western University, Langley, Canada; 3 Faculty of Medicine, University of British Columbia, Vancouver, Canada

**Keywords:** Endurance-trained, Cardiac function, Arterial compliance, Left ventricular mass, Longitudinal strain rate, VO_2_max

## Abstract

**Background:**

Aerobic capacity measured by maximal oxygen uptake (VO_2_max) is related to functional capacity and is a strong independent predictor of all-cause and disease-specific mortality. Sex-specific cardiac and vascular responses to endurance training have been observed, however, their relative contributions to VO_2_max are less understood. The purpose of this study was to evaluate sex-specific ventricular-vascular interactions associated with VO_2_max in healthy males and females.

**Methods:**

Sixty-eight males and females (38% females, 35 ± 10y) characterised as recreational exercisers to highly trained endurance athletes, and free of chronic disease underwent a cycle ergometer to assess VO_2_max. Resting arterial compliance and echocardiographic evaluation of left ventricular (LV) structure and function were measured and indexed to body surface area.

**Results:**

VO_2_max was similar between groups (54 ± 6 vs. 50 ± 7 ml/kg/min, *p* = 0.049). Indexed LV mass (LVMi) was higher (96 ± 15 vs. 81 ± 11, *p* = 0.001) in males versus females, respectively. Linear regression analysis revealed two models that were significantly associated with VO_2_max in males and females. In males, the two models included (1) longitudinal diastolic strain rate and LVMi (r^2^ = 0.31, *p* = 0.003) and (2) indexed end-diastolic volume (EDVi) and longitudinal diastolic strain rate (r^2^ = 0.34, *p* < 0.001). In females, the linear regression models included (1) LVMi, large arterial compliance, longitudinal systolic strain rate, and age (r^2^ = 0.69, *p* < 0.001) and (2) EDVi, large arterial compliance, longitudinal systolic strain rate, and age (r^2^ = 0.52, *p* = 0.003).

**Conclusion:**

These findings reveal that while in both sexes, LVMi and LVEDVi are associated with VO_2_max, arterial compliance was also found to contribute to the variance in VO_2_ max in females, but not in males. Further, ventricular relaxation was a significant factor in aerobic capacity in males, while in females ventricular contraction was a significant factor.

## Introduction

Regular endurance training enhances cardiac structure and function so that the increased demands of exercise can be met via increased cardiac output [1–3]. Given that maximal heart rate is not enhanced by endurance training, any increase in maximal cardiac output (CO) is manifested by increases in stroke volume (SV) [4, 5]. There is also an expansion of plasma volume with endurance training which facilitates venous return leading to higher end-diastolic volume (EDV) and SV [6]. Factors associated with endurance-trained SV augmentation include increased ventricular mass, increased EDV, more rapid diastolic filling and enhanced systolic contractility [4, 7]. Echocardiographic 2D speckle tracking can identify subtle physiological differences in adaptations to cardiac mechanics such as global longitudinal strain, which is positively associated with left ventricular mass (LVM) and differs between athletes and healthy controls [8]. The arterial system also plays an important role in increased CO with training through vascular adaptions (i.e., increased artery diameters and decreased wall thickness) that increase arterial compliance supporting enhanced delivery of oxygenated blood to exercising muscle [6]. Together, these central and peripheral factors largely contribute to maximal aerobic capacity (i.e., VO_2_max); in fact, 70–85% of the variance in VO_2_max can be explained by cardiac output alone [9, 10].

Sex-specific differences in cardiac structure and function have been observed in response to endurance training. In a recent meta-analysis, LVM indexed to body surface area was found to be similarly augmented following endurance training in both sexes [11]. However, while both males and females showed augmented LV cavity size, an increase in LV wall thickness was observed only in males [11]. Further, endurance training-induced LVEDV appears to increase to a greater extent in males compared to females with left ventricular SV enhanced only in males [12]. By contrast, cardiac function has been shown to be reduced to a greater extent in males following high-intensity interval exercise and prolonged endurance exercise than in females [13, 14], which may indicate greater sensitivity to changes in contractility in response to strenuous exercise than females. Taken together, there appears to be substantive evidence showing that while there are adaptive cardiac responses in both males and females as a result of endurance training, these patterns of functional adaptation are unique for each sex.

Arterial remodelling is also a positive consequence of endurance training. Endurance training induces episodic increases in arterial shear stress, which triggers endothelium-mediated remodelling in the conduit arteries as they adapt to higher volume demands [15]. This remodelling process leads to arterial enlargement, optimizing blood flow and facilitating improved oxygen and nutrient delivery to the muscles during exercise [16]. This enhanced arterial compliance, as a result of arterial remodelling, appears to be greater in males compared to females in both endurance-trained individuals and also previously inactive individuals [17].

Relative ventricular-vascular contributions to VO_2_max may differ by sex due to sex differences in cardiac and vascular properties, and associations between arterial stiffness or compliance with exercise cardiac mechanics [13, 18, 19]. Therefore, the purpose of this study was to assess sex-specific ventricular-vascular interactions associated with aerobic capacity in healthy males and females. We hypothesized there would be a greater vascular contribution to aerobic capacity in females, whereas in males there would be a stronger association between ventricular function and aerobic capacity.

## Methods

### Participants

A convenience sample of healthy male and female adults were recruited from the local community. Inclusion criteria consisted of meeting the minimum physical activity guidelines of 150 min of moderate to vigorous aerobic physical activities per week [20] (e.g. normally active to endurance-trained). Participants were excluded if they had any underlying chronic health conditions and/or were current smokers. This research was approved by Trinity Western University Research Ethics Board and all participants provided written informed consent.

### Experimental protocol

Participants completed a pre-screening (Physical Activity Readiness Questionnaire for Everyone (PAR-Q+) [21], a training and medical history questionnaire, and were measured for body height (m) and mass (kg, Seca 869, USA). Body mass index (BMI) was calculated as kg /m^2^, and body surface area (BSA) was calculated using the Mosteller Formula [22]. The medical questionnaire included a section for females asking about contraception and menstrual cycle stage on test day. Vascular measures (resting blood pressure, arterial compliance), preceded echocardiography. To assess maximal aerobic capacity we measured VO_2_max, characterized as the highest rate of O_2_ uptake measured in the final minute during a single maximum test [23]. VO_2_max assessments were obtained following the resting assessments on the same or subsequent day given participant availability. All participants were instructed to abstain from caffeine and alcohol for 12 h and strenuous exercise 48 h prior to each testing day.

### Aerobic capacity

An incremental bike test to exhaustion (Quark Velotron, SRAM, Chicago, USA) was used to assess VO_2_max [17]. Participants started the test at 80–100 watts, increasing workload by 25 watts every 2 min, pedalling at 80–90 revolutions per minute. Oxygen consumption was measured from expired gases analysed by a calibrated metabolic cart (TrueOne 2400, Parvo Medics, Salt Lake City, USA). Heart rate (Polar, Kempele, Finland), and ratings of perceived exertion using the original 20 point Borg scale [24] were recorded throughout the test. Participants were encouraged to cycle to volitional fatigue. Maximal aerobic capacity was reached if respiratory exchange ratio > 1.1, peak heart rate was within 95% of age-predicted maximum, and cadence dropped below 80 rpm and could not be recovered (i.e., reached volitional fatigue).

### Vascular measurements

Baseline vascular measurements were assessed after 10 min of rest in in the supine position. Two automated blood pressure measurements were obtained by brachial occlusion (BpTRU 100, Coquitlam, Canada) and averaged. Arterial compliance was measured non-invasively via applanation tonometry with the HDI CR-2000 (Hypertension Diagnostics, Eagan, MN) for diastolic pulse contour analysis. This method of vascular assessment using waveform shape analysis is considered optimal for measuring systemic compliance [25] and is based on a modified Windkessel model that allows for the estimation of large (capacitive) artery and small (oscillatory) artery compliance (LAC and SAC, respectively). The right wrist was stabilized with the automated sphygmomanometer affixed to the upper left arm. Maximal signal strength was obtained prior to the radial artery tonometry measurement. Two measurements were taken and the average was used for analysis. Total peripheral resistance (TPR) was calculated by dividing the mean arterial pressure (MAP) by the cardiac output (CO, TPR = MAP/CO), where MAP adds diastolic blood pressure (DBP) to one-third pulse pressure (PP) (systolic blood pressure (SBP) – diastolic blood pressure) (MAP = 1/3(PP) + DBP.

### Echocardiography

Echocardiographic image acquisition was performed by a clinical sonographer accredited by the American Registry for Diagnostic Medical Sonography (ARDMS), using a portable ultrasound unit (Vivid iq, GE Medical Systems, USA) with simultaneous ECG and a 2.5-MHz transducer. Participants were positioned on the treatment table in the left lateral decubitus position for imaging. A parasternal long-axis view was acquired for the measurement of LV dimensions and mass, while apical two- and four-chamber views were acquired for the assessment of LV volumes and function [26, 27]. Transmitral Doppler peak flow velocities were measured for early (E) and late (A) diastolic filling, and myocardial tissue velocities were assessed at the mitral annulus at the LV septal (E’ septal), and LV lateral (E’ lateral) walls. Three consecutive cycles were recorded for each.

Ventricular volumes, structure and function were analysed offline (EchoPAC, GE Healthcare, v203) by one researcher with extensive experience in echocardiographic analysis (AC). Left ventricular mass was determined using the linear method and the Cube formula and the units used in the equation were in centimetres [26]. Relative wall thickness (RWT) was calculated using the Eq. 2 x posterior wall thickness (PWT) /LV end-diastolic diameter (LVDd). RWT and indexed LVM (LVMi) were plotted as a scatterplot to illustrate LV geometry for normal, concentric remodelling, eccentric remodelling and concentric hypertrophy according to current recommendations [26].

Left ventricular volumes were determined using the Simpson’s bi-plane method. The product of SV ((EDV) – end systolic volume (ESV)) and heart rate, CO, and LVM were indexed to body surface area (SVi, COi, and LVMi, respectively). Ejection fraction (EF) was calculated as SV as a percentage of EDV. Left ventricular wall stress was calculated as 0.133 x P x R/2T x (1 + T/2*R*), where P is systolic blood pressure, *R* is LV end-systolic diameter, and T is LV systolic posterior wall thickness [28]. A surrogate of left ventricular contractility was calculated from the ratio of systolic blood pressure and end-systolic volume (SBP/ESV) [29]. Diastolic function was assessed by calculating E/A ratio, E/E’ septal and E/E’ lateral.

Speckle tracking analysis was used to assess LV global longitudinal strain and systolic and diastolic strain rates. LV longitudinal strain and strain rate were derived using speckle-tracking analysis using the apical four-chamber, two-chamber and three-chamber views. The region of interest was adjusted to include the whole myocardium. Automated strain analysis provided peak strain data for six myocardial segments for each of the three views and an average of these provided a global value of the 18 segments. All strain imaging was acquired at high frame rates (80–90 frames/second) and adjustments were made during image acquisition to provide clear image quality.

### Statistical analysis

Data was stratified by sex and checked for normality. Independent sample T-tests were used for continuous variables to determine sex-based differences for vascular and left ventricular structure and function and chi-square tests were used for categorical variables (i.e., LV geometry). Bonferroni correction was applied to the independent T-tests given multiple number of comparisons and alpha was set at *p* ≤ 0.001. Simple bivariate correlations were performed using Pearson correlation. A between-group analysis of variance (ANOVA) was used to assess the effect of geometry on VO_2_max, with post hoc analysis conducted using Scheffe’s test. Stepwise linear regression was performed and included age, and ventricular and vascular variables for each sex to address our research question and significant variables identified with the Pearson correlation. Collinearity was considered in all models. Intra- and inter-observer variability was assessed by selecting 10 studies that were blindly randomized by a separate investigator and quantified using intraclass correlation coefficients (ICC) and 95% confidence intervals (CI). Significance was set at *p* < 0.05. All analyses were performed using SPSS software (version 29.0; SPSS IBM, Chicago, IL) and data was presented as mean ± standard deviation.

Inter-rater ICCs were (blindly) performed between a BSE accredited sonographer and a clinical sonographer accredited by ARDMS, and subsequently between AC and the ARDMS accredited sonographer and was reported as our lab’s inter-rater ICCs and AC performed the intra-rater ICCs.

## Results

**Fig. 1:**
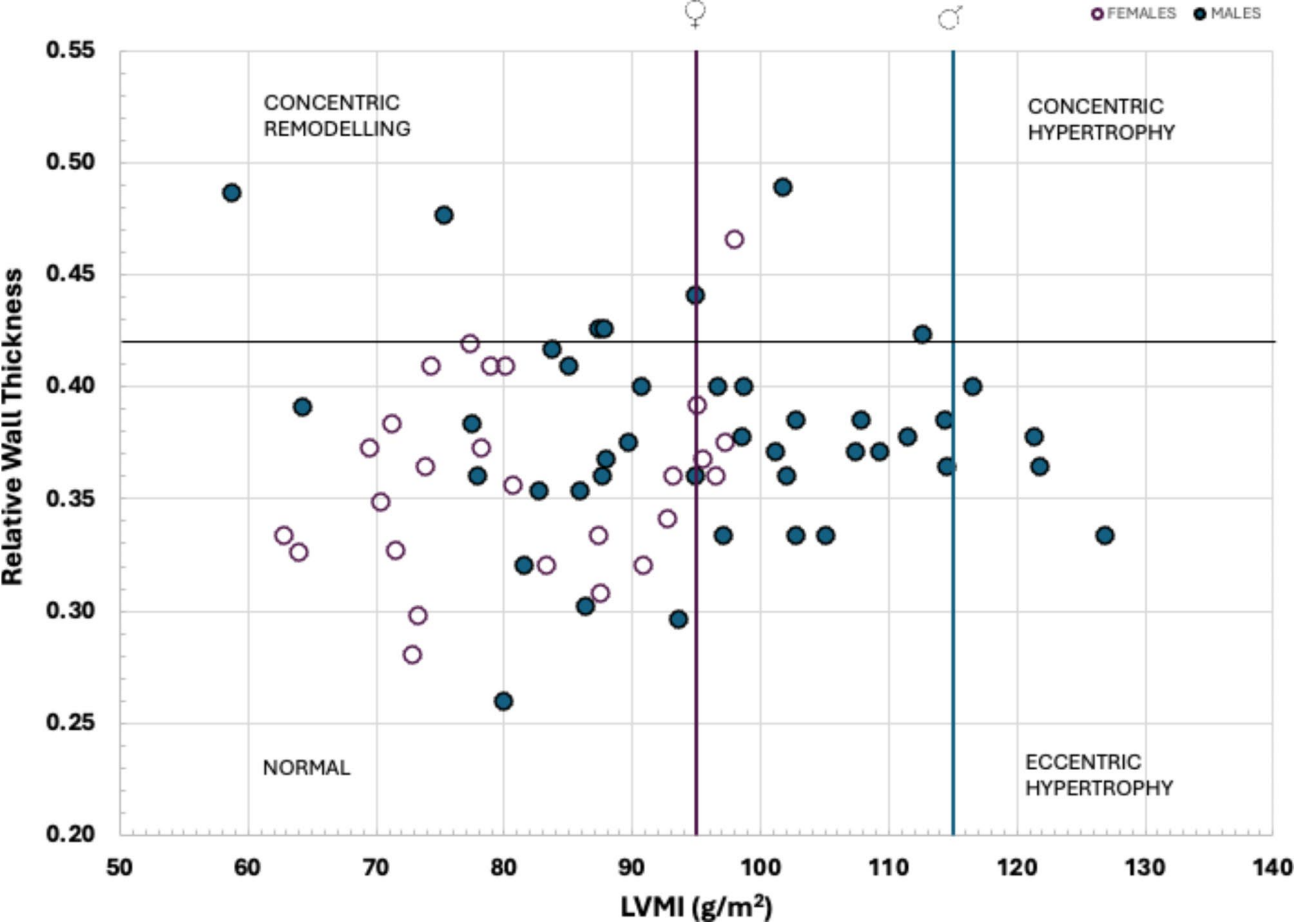
Left ventricular (LV) geometry illustrated by the relationship of indexed left ventricular mass (LVMi) and relative wall thickness (RWT) using upper limit cut-offs for LVMi: males ≤115 g/m^2^ (open circles) and females ≤95 g/m^2^ (green circles) and RWT, ≤42 according to current guidelines [26]

**Fig. 2:**
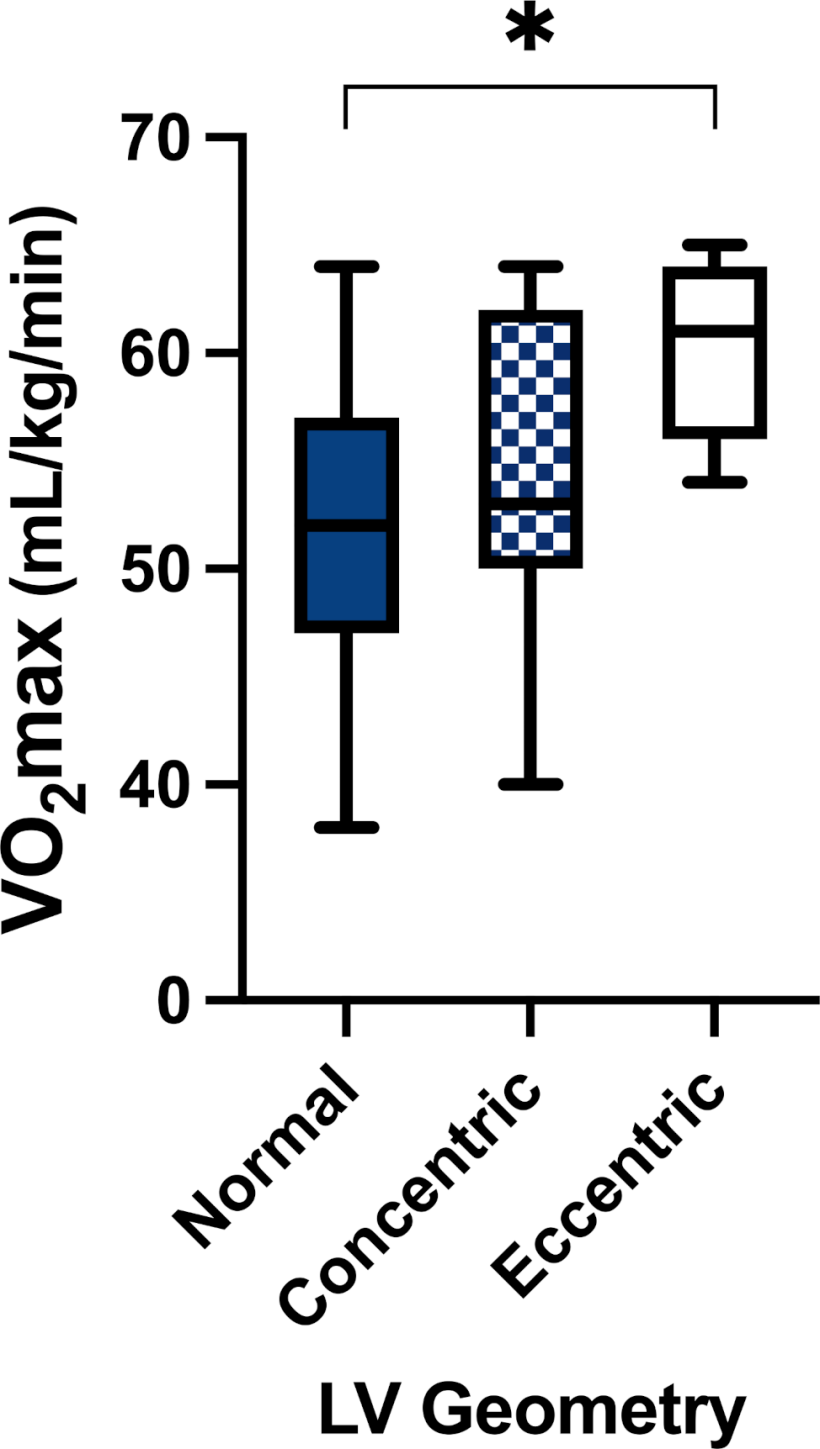
VO_2_max according to left ventricular geometry (mean ± standard deviations) for the entire sample as sex differences in geometry were not observed (*p* = 0.385). Concentric represents both concentric hypertrophy and concentric remodeling. The eccentric hypertrophy group had significantly higher VO2max compared with Normal (*p* = 0.002) but did not differ with Concentric (*p* = 0.202). Normal was also not statistically different from Concentric (*p* = 0.751)

A total of 68 participants were assessed in this study (38% females; Table 1); the population was predominately of European descent (97%). Participants reported engaging in a variety of exercise activities that ranged from recreational pursuits (i.e., daily bike commuting, aerobic fitness classes, recreational running) to high-volume endurance training (competitive triathletes, cyclists, runners, cross-country skiers). 35% (*n* = 10) of the females reported being on hormonal contraception (*n* = 2 intrauterine device; *n* = 8 oral contraceptives). Of those with a natural menstrual cycle, 60% were assessed in the follicular phase and 40% in the luteal phase. Age was not different between groups and BMI was in the normal range. Mean SAC was lower in females (8±3 vs. 10±3 mm Hg x 100^− 1^, *p* < 0.001). VO_2_max ranged from 40 to 65 mL/kg/min (53.8 ± 6.4) and 38–63 mL/kg/min (50.5 ± 7.0) for males and females (*p* = 0.049), respectively, but did not reach statistical significance after the Bonferroni correction (p *≤* 0.001). Sex differences were evident for LVM, IVSd, intraventricular septum during systole (IVSs), PWTd, PWTs, LVDd, EDV, SV, CO, however, once indexed to BSA, these differences were no longer significant (p *≥* 0.001) except for LVMi (Table 2). Sex differences in cardiac function did not meet the threshold for statistical significance (p *≥* 0.001). The results of LV geometry are shown in Fig. 1. The majority of females (85%) and males (76%) had normal geometry. None of the males elicited concentric hypertrophy, while there was one (4%) female who did. Six males (14%) elicited concentric remodelling, while none of the females did. Four males (10%) and three females (11%) had eccentric hypertrophy. Proportions allocated by geometry type did not differ by sex (*p* = 0.385). As a group, VO_2_max was statistically greater in those with eccentric remodelling than those with normal geometry (*p* = 0.002), but there was no statistical difference in VO_2_max between normal and concentric remodelling/hypertrophy (*p* = 0.751) nor between concentric remodelling/hypertrophy and eccentric hypertrophy groups (*p* = 0.202, Fig. 2).

**Table 1:** Participant characteristics and vascular indices

	Males (*n* = 42)	Females (*n* = 26)	*p*-value
Age, y	36 ± 10	34 ± 10	0.411
Height, cm	179 ± 7	165 ± 6	**< 0.001**
Weight, kg	76 ± 8	60 ± 9	**< 0.001**
BMI, kg/m^2^	24 ± 2	22 ± 2	0.002
BSA, m^2^	1.9 ± 0.1	1.7 ± 0.1	**< 0.001**
Heart rate, bpm	55 ± 9	57 ± 8	0.312
SBP, mmHg	116 ± 13	108 ± 13	0.010
DBP, mmHg	72 ± 10	69 ± 8	0.126
LAC, mL/mmHg^x10^	20 ± 6	17 ± 4	0.053
SAC, mL/mmHg^x100^	10 ± 3	8 ± 3	**< 0.001**
VO_2_max, mL/kg/min	54 ± 6	50 ± 7	0.049

BMI = body mass index; BSA = body surface area; SBP = systolic blood pressure; DBP = diastolic blood pressure; LAC = large arterial compliance; SAC = small arterial compliance

**Table 2:** Cardiac structure and function

	Males (*n* = 42)	Females (*n* = 26)	*p*-value
**Dimensions and Mass**			
LVM, g	186 ± 30	134 ± 20	**< 0.001**
LVMi, g/m^2^	96 ± 15	81 ± 11	**0.001**
IVSd, mm	10.2 ± 0.9	8.9 ± 0.9	**< 0.001**
IVSid, mm/m^2^	5.3 ± 0.5	5.4 ± 0.6	0.544
PWTd, mm	9.5 ± 0.8	8.3 ± 0.7	**< 0.001**
PWTid, mm/m^2^	4.9 ± 0.5	5.0 ± 0.6	0.298
LVDd, mm	50.5 ± 4.2	46.4 ± 3.0	**< 0.001**
LVDid, mm/m^2^	26.1 ± 2.7	28.1 ± 2.2	0.002
IVSs, mm	17.8 ± 2.0	15.5 ± 2.0	**< 0.001**
IVSis, mm/m^2^	9.2 ± 1.1	9.4 ± 1.2	0.520
PWTs, mm	17.1 ± 2.1	15.0 ±2.3	**< 0.001**
PWTis, mm/m^2^	8.8 ± 1.1	9.0 ± 1.4	0.419
LVDs, mm	30.0 ± 4.6	27.3 ± 3.5	0.019
LVDis, mm/m^2^	15.4 ± 2.3	16.5 ± 2.0	0.044
RWT	0.38 ± 0.05	0.36 ± 0.04	0.098
**Volumes and Basic Hemodynamics**			
EDV, mL	149 ± 32	110 ± 27	**< 0.001**
EDVi, mL/m^2^	77.0 ± 16.0	66.4 ± 14.5	0.008
ESV, mL	54 ± 18	41 ± 16	0.003
ESVi, mL/m^2^	15.4 ± 2.3	16.5 ± 2.0	0.167
SBP/ESV	3.7 ± 1.3	4.5 ±1.7	0.041
SV, mL	95 ± 22	69 ±16	**< 0.001**
SVi ml/m^2^	49 ± 11	42 ± 8	0.005
EF, %	64 ± 8	63 ± 8	0.816
CO, L/min	5.3 ± 1.3	4.0 ± 1.0	**< 0.001**
Ci, L/min/m^2^	2.8 ± 0.7	2.4 ± 0.6	0.029
TPRi, mmHg/min	8.6 ± 2.6	12.5 ± 4.3	**< 0.001**
Wall stress, g/cm^2^	146 ± 51	89 ± 25	**< 0.001**
**Systolic and Diastolic Function**			
Longitudinal strain, %	-20 ± 2	-20 ± 2	0.782
Systolic longitudinal strain rate, s^− 1^	-1.0 ± 0.1	-1.0 ± 0.1	0.714
Diastolic longitudinal strain rate, s^− 1^	1.4 ± 0.2	1.6 ± 0.3	0.005
E, m/s	0.8 ± 0.2	0.8 ± 0.2	0.313
A, m/s	0.5 ± 0.1	0.4 ± 0.1	0.357
E/A ratio	1.8 ± 0.4	2.1 ± 0.6	0.036
DecT, ms	223 ± 51	209 ± 35	0.201
E’ septal wall, m/s	0.13 ± 0.03	0.13 ± 0.02	0.789
E/E’ septal	5.9 ± 2.2	6.7 ± 1.5	0.093
E’ lateral wall, m/s	0.18 ± 0.03	0.17 ± 0.03	0.340
E/E’ lateral	4 ± 1	5 ± 1	0.018
IVRT, m/s	80 ± 12	77 ± 14	0.259

Note: Dimensions, mass and volume measures are shown as both absolute values and relative values (indexed to body surface area); Dimensions are shown obtained in systole (s) and diastole (d)

LVM = left ventricular mass, IVS = intraventricular septal wall thickness, PWT = posterior wall thickness, LVD = left ventricular diameter, RWT = relative wall thickness, E = early diastolic filling, A = late diastolic filling, DecT = deceleration time, E’ = tissue velocity, IVRT = isovolumetric relaxation time

**Fig. 3:**
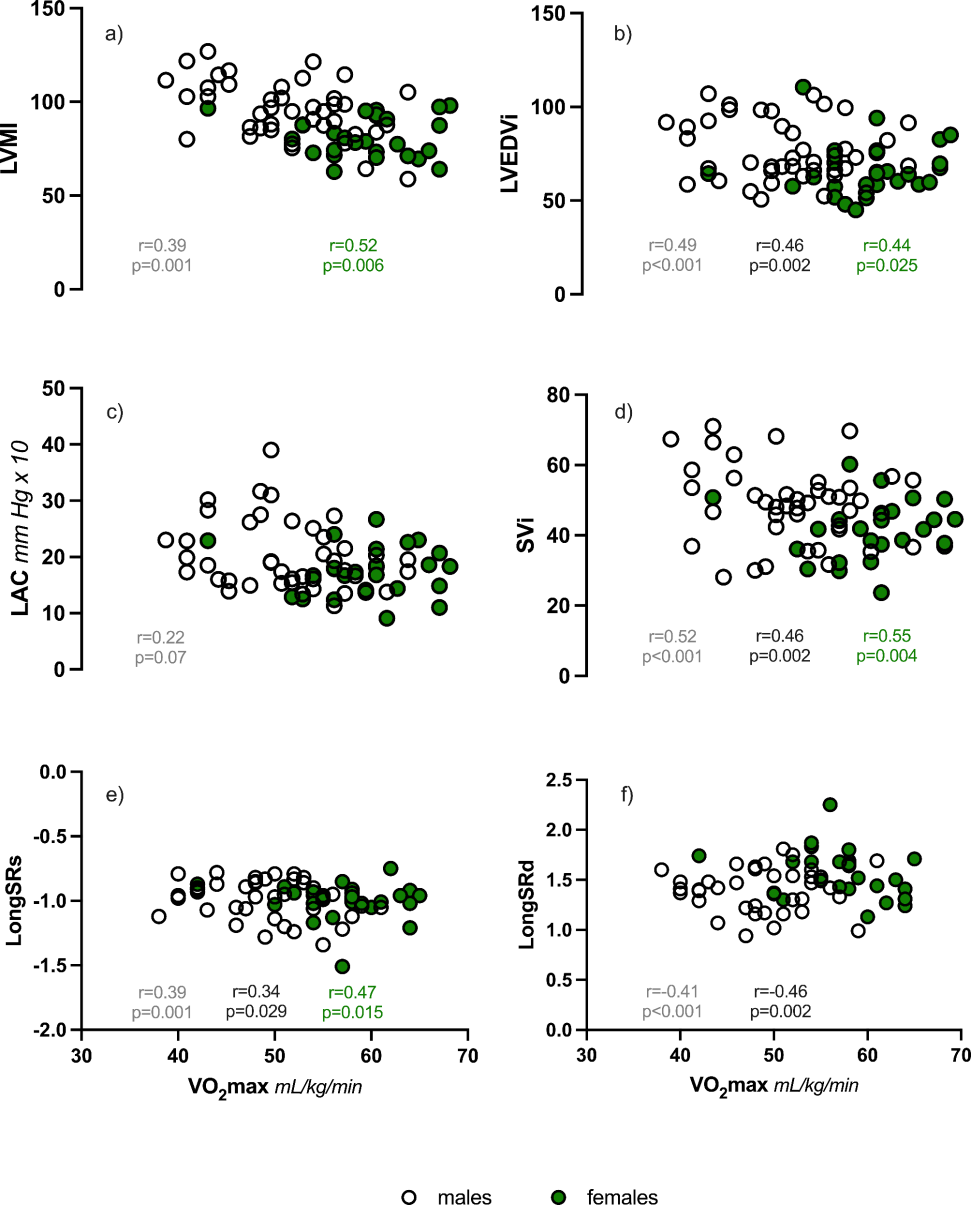
Independent associations between VO_2_max and **(a)** left ventricular mass index (LVMi); **(b)** end-diastolic volume index (EDVi); **(c)** large arterial compliance (LAC); **(d)** stroke volume index (SVi); **(e)** longitudinal systolic strain rate (LongSRs); and **(f)** longitudinal diastolic strain rate (LongSRd) in males (open circles) and females (green circles). Statistically significant correlation coefficients are shown for the entire population (grey), males (black), and females (green)

Bivariate correlations that significantly correlated with VO_2_max were heart rate (*r* = -0.374, *p* = 0.015), EDVi (*r* = 0.462, *p* = 0.002), SVi (*r* = 0.464, *p* = 0.002), longitudinal systolic strain rate (*r* = 0.337, *p* = 0.029), and longitudinal diastolic strain rate (*r* = -0.459, *p* = 0.002) in males and LVMi (*r* = 0.524, *p* = 0.006), EDVi (*r* = 0.439; *p* = 0.025), SVi (*r* = 0.548; *p* = 0.004), wall stress (*r* = 0.421, *p* = 0.032), and longitudinal systolic strain rate (*r* = 0.477, *p* = 0.015) in females. Figure 3 illustrates the independent relationships between key variables and VO_2_max. The linear regression analysis revealed the variables associated with VO_2_max in two unique models for each sex (Table 3). In males, the two models included (1) longitudinal diastolic strain rate and LVMi (r^2^ = 0.31, *p* = 0.003) and (2) EDVi and longitudinal diastolic strain rate (r^2^ = 0.34, *p* < 0.001). In females, the linear regression models included (1) LVMi, LAC, and longitudinal systolic strain rate (r^2^ = 0.69, *p* < 0.001) and (2) EDVi, LAC, and longitudinal systolic strain rate (r^2^ = 0.52, *p* = 0.003).

**Table 3:** Determinants of VO_2_max in healthy, active males and females

	*r* ^2^	Std. β	95% confidence intervals	*p*-value
**Males**				
**Model 1**	0.31			< 0.003
LVMi		0.316	0.017, 0.247	0.024
LongSRd		-0.514	-24.080, -7.087	< 0.001
Age		-0.038	-0.207, 0.161	0.801
**Model 2**	0.35			< 0.001
LVEDVi		0.381	0.041, 0.262	0.008
LongSRd		-0.365	-19.51, -2.60	0.012
Age		0.029	-0.163, 0.201	0.831
**Females**				
**Model 1**	0.69			< 0.001
LVMi		0.565	0.197, 0.525	< 0.001
LAC		0.559	0.348, 1.448	0.003
LongSRs		0.443	9.036, 34.474	0.002
Age		0.469	0.090, 0.554	0.009
**Model 2**	0.52			0.003
LVEDVi		0.396	0.032, 0.349	0.021
LAC		0.451	0.047, 1.403	0.037
LongSRs		0.392	3.275, 35.214	0.021
Age		0.527	0.072, 0.653	0.017

LVMI = left ventricular mass index; LongSRd = longitudinal diastolic strain rate; LongSRs = longitudinal systolic strain rate; LVEDVi = left ventricular end-diastolic volume index; LAC = large arterial compliance

The lab’s inter-rater ICCs (95%CI) for the measurement of LV dimensions are IVSd: 0.890 (0.790–0.954), LVDd: 0.910 (0.822–0.980), and PWTd: 0.906 (0.884–0.934). Intra-rater ICCs (95% CI) are IVSd: 0.900 (0.824–0.966), LVDd: 0.926 (0.904–0.944) and PWTd: 0.950 (0.922–0.980). Inter-observer ICCs (95% CI) are 0.90 (0.77, 0.97), 0.92 (0.68, 0.98) and 0.92 (0.82, 0.98) for longitudinal strain, systolic strain rate, and diastolic strain rate, respectively. Intra-observer ICCs (95% CI) are 0.99 (0.94, 0.99), 98 (0.96, 0.98) and 0.98 (0.96, 0.99) for longitudinal strain, systolic strain rate, and diastolic strain rate, respectively.

## Discussion

In this study, we assessed sex-specific ventricular-vascular interactions associated with aerobic capacity in healthy males and females. While LVMi and LVEDVi was positively associated with aerobic capacity in both sexes, age and arterial compliance were also associated with aerobic capacity in females. Further, we found distinct differences in strain rate contribution to VO_2_max with diastolic strain rate (ventricular relaxation) a significant factor in males and systolic strain rate (ventricular contraction) a significant factor in females.

### Vascular factors associated with aerobic capacity

Our results show an increased reliance on vascular adaptations in females with higher aerobic capacity. It has been previously demonstrated that athletes possess larger conduit arteries than non-athletes [15] and athletes possess greater arterial compliance [13, 17, 30] and endurance training increases arterial compliance in previously untrained adults [31–34]. With endurance training, healthy arteries remodel in response to increased volume demands, resulting in an enlargement of lumen diameter and reduction in wall thickness [15]. These adaptations lead to enhanced arterial compliance [35]. We have previously illustrated the relationship between large and small artery compliance and aerobic capacity (VO_2_max) in both males and females of a wide training status range (inactive to endurance-trained) [17]. The results of our present investigation extend our previous findings by modelling the additional factors that contribute to maximal aerobic capacity in athletic individuals vis-à-vis biological sex.

### Are female conduit arteries more amenable to exercise?

A novel finding in this study is that arterial compliance is predictive of VO_2_max in females but not in males. This prompts discussion about whether the female arterial system is relatively more amenable to endurance exercise. For example, it is well known that central elastic arteries buffer high pressures and flow [36], yet little is known about sex differences in the adaptive response to exercise on the arterial system.

It is well known that premenopausal females exhibit a reduced risk of cardiovascular disease compared to males likely attributed to the cardioprotective effect of oestrogen [37]. However, testosterone has also been shown to have protective effects on blood vessels [38], influencing vascular reactivity [39] and arterial compliance in males [40]. Hormone receptors are expressed in the vasculature [38, 41] with both oestrogen and testosterone improving vascular function through enhanced endothelial function while testosterone also improves smooth muscle cell function [42]. However, nitric oxide (NO) production is greater in premenopausal women than in men and females appear to be more sensitive to NO-mediated vasodilatory effects of oestrogen [43]. Given exercise training promotes improvement in endothelial function through the arterial shear stress exhibited during exercise [44], the cumulative effects of increased NO availability and exercise in females may result in a relative greater improvement in arterial compliance compared to that of males. Indeed, hormone fluctuations during the menstrual cycle have been reported to influence whole body arterial compliance [45, 46]. Since reproductive hormones play an important role in vascular function, the association with age may be a reflection of this. It should be noted that to date, there are no studies specifically examining the role of sex hormones on changes in arterial compliance with chronic endurance exercise.

### Cardiac factors associated with aerobic capacity

Our data shows cardiac adaptations are associated with aerobic capacity in both males and females. Previous studies have assessed structural and functional cardiac measures in relation to aerobic capacity with differing outcomes depending on the variables investigated and the imaging modality utilized. For example, using cardiac magnetic resonance imaging (CMR) and Doppler echocardiographic strain, La Gerche et al. [47] reported LVMi, right ventricular EDVI, and heart rate reserve were the strongest predicters of VO_2_max in a predominately male sample (49 males, 6 females). They concluded that structural rather than functional measures enabled the greatest oxygen consumption. When they analysed the males and females separately, the predictors of VO_2_max remained the same in the males, while in females, only LVMi was associated with VO_2_max. Vascular measures were not included in their analysis. In another CMR-based study, Swoboda et al. [48] assessed LVMi, LVEDVi, right ventricular EDVI and bi-ventricular strain in 35 endurance athletes (77% male), but found only RV longitudinal late diastolic strain rate had a significant association with VO_2_max. While both studies involved predominately male participants, La Gerche’s study included non-athletes and is more aligned with our sample. However, they did not use speckle track imaging or CMR derived strain which could explain why they did not find an association with diastolic strain rate as found in our study and that of Swoboda et al. [48].

Endurance training stimulates ventricular remodelling and improves LV relaxation and compliance which ultimately enhances LV filling capacity and SV required for improvements in cardiorespiratory fitness [49], which explains why LVMi and LVEDVi was a significant predictor in both sexes and longitudinal diastolic strain rate was a predictor in males. Interestingly, longitudinal diastolic strain rate was not a predictor in females, however, longitudinal systolic strain rate which is a marker of ventricular contraction was. While speculative, the contribution of longitudinal systolic strain rate in females could be a compensatory mechanism to ensure adequate cardiac output, given the generally smaller female heart (i.e., lower LV volumes). Oestrogen has also been proposed to influence better systolic function in females [50] and age-related decreases in longitudinal strain have been identified in females but not males [51]. Thus, given the age range of our female athletes and the association of age in our statistical models, age and/or hormonal differences between males and females are potential factors responsible for the increased contribution of longitudinal systolic strain rate in females and warrants further study.

Endurance training elicits cardiac remodelling which can be categorized into four groups of LV geometry (normal, concentric remodelling, concentric hypertrophy and eccentric hypertrophy), with most athletes exhibiting normal geometry [52, 53]. Previous work by Oxborough et al. [52] examined LV geometry in both sexes and of a similar mean age to our study. These findings of Oxborough et al. [52] align with the present study where there was a low prevalence of concentric hypertrophy in males and females and a similar prevalence of eccentric hypertrophy in males (8.3% vs. 10%) and females (9.2% vs. 11%). More males elicited concentric remodelling in the present study compared to Oxborough et al.’s study (14% vs. 7.7%, respectively), however, were similar to Finocchiaro et al. who reported 15% of male athletes had concentric remodelling/hypertrophy [53]. It is widely known that cardiac remodelling is related to age, sex body surface area, and type of sport participated, but other factors such as increased blood pressure at rest and during exercise, body weight, and body fat percentage can also be contributors [55]. While the average resting blood pressures in males were within normal limits in the present study, there were some that had elevated resting blood pressures and could have contributed to the increased concentric remodelling in the present study. Resting blood pressures were not reported in Oxborough et al.’s study, and neither the present study nor Oxborough et al.’s study studied blood pressure responses during exercise, therefore we cannot be certain that this is what contributed to the elevated concentric remodelling in the present study, but is a possible explanation that could explain the difference.

While the observed variance in VO_2_max is moderate (31–35% in males and 52–69% in females) other factors could have accounted for VO_2_max that we did not directly assess. For example, increased red blood cell volume, which will affect a-vO_2diff,_ and augmentation in capillarization and mitochondrial content which contributes to O_2_ extraction [54], cannot be accounted for in our study. These adaptations are usually present after 12 weeks of training, therefore all participants in our study should be impacted by these factors, however, their relative contributions based on sex warrant consideration in future studies.

### Limitations

In the present study we did not assess the right ventricle which may have presented additional contributing factors not identified with only considering the left ventricle. Furthermore, it is possible that measures of cardiac function assessed during exercise could explain more of the variance in VO_2_max that we are not able to identify during resting echocardiography. We did not scale our data allometrically as our population was too small to derive adequate allometric exponents, as such, we scaled ratiometrically as this is currently routine clinical practice. The present population was predominately white, and therefore, we were unable to consider the impact of ethnicity/race in our results. Lastly, the menstrual cycle was not controlled for as our intention was to find robust contributors to aerobic capacity that can be attributed to training, not cycle fluctuations. However, the degree to which hormonal fluctuations may influence the strength of the association with aerobic capacity could be explored in future research.

## Conclusion

In active and endurance-trained males and females, we found that LVMi and LVEDVi were significant contributors to VO_2_max in both sexes, with additional sex-specific factors explaining the variance in aerobic capacity. Longitudinal diastolic strain rate, a measure of ventricular relaxation was identified as a significant factor in males, while arterial compliance, longitudinal systolic strain rate and age were significant factors in females. These sex-specific ventricular and vascular contributions to aerobic capacity in male and female active and endurance-trained are novel and require further study.

## Data Availability

The datasets used and/or analysed during the current study are available from the corresponding author on reasonable request.
